# Reducing PTV margins for prostate SBRT with motion compensation and gating techniques

**DOI:** 10.1002/acm2.13861

**Published:** 2022-12-07

**Authors:** Lee Goddard, Kyoungkeun Jeong, Justin Tang, Madhur Garg, Wolfgang A. Tomé

**Affiliations:** ^1^ Department of Radiation Oncology Montefiore Medical Center Bronx New York USA; ^2^ Albert Einstein College of Medicine Bronx New York USA

**Keywords:** Prostate SBRT intrafraction motion

## Abstract

The purpose of this study is to investigate the dosimetric accuracy of prostate SBRT when motion is considered. To account for target movement, motion compensation and gating techniques were investigated with PTV margins reduced to 2 mm. To allow for dosimetric measurements a Delta4 phantom, Gafchromic film, and Hexamotion motion platform were utilized. Four motion files were utilized that represent a range of motions. Analysis of measured prostate motions for fifteen patients was performed to ensure detected motions were similar to those previously reported and motion files utilized were suitable. Five patient plans were utilized to allow for the effects of MLC and target motion interplay to be investigated. For both motion compensation and gating techniques, plans were delivered to the stationary phantom and for each of four motion types with/without compensation/gating enabled. Using a 3%, 2 mm and 80% threshold gamma criteria, film measurements had an average pass rate of 80.5% for uncorrected deliveries versus 96.0% for motion compensated deliveries. For gated techniques average pass rates increased from 89.9% for uncorrected to 94.8% with gating enabled. Measurements with the Delta4 arrays were analyzed with a 3%, 2 mm and 10% threshold dose. An average pass rate of 83.8% was measured for uncorrected motions versus 94.8% with motion compensation. For the gated technique an average pass rate of 87.2% was found for uncorrected motions versus 96.9% with gating enabled. These results show that very high gamma pass rates are achievable when motion compensation or gating techniques are applied. When target motion is not accounted for shifts up to 5 mm in planned versus delivered isodose distributions were found. However, when motion compensation, or gated techniques were applied, much smaller differences between planned and delivered isodose distributions were found. With these techniques dose delivery accuracy is greatly improved, allowing for PTV margins to be reduced.

## 1 INTRODUCTION

PTV margins for prostate radiotherapy are highly variable depending on the imaging and immobilization techniques used.^1^ It is desirable to reduce the PTV margins wherever possible by utilizing more accurate treatment delivery techniques, but it is especially critical in Stereotactic Body Radiation Therapy (SBRT). Radiobiological evidence indicates that the alpha/beta ratio of prostate cancer could be as low as 1.5 Gray (Gy),^2^, ^3^, ^4^ supporting the use of a hypofractionated dose regimen. SBRT allows for the precise deposition of dose required for these hypofractionated treatments and is becoming increasingly common.^5^, ^6^

Even if a patient remains perfectly still during treatment setup and delivery, the prostate moves naturally within the body due to bladder filling and transient gas in the rectum. The PTV margin for prostate cancer can be significantly reduced by limiting the target motion or by tracking the target during treatment delivery to either pause the treatment when the target moves by an excessive amount, known as gating, or by adjusting the delivery to account for target motion.

Litzenberg et al. demonstrated a reduction in PTV margin to less than 2 mm is possible if intrabeam adjustments or continuous tracking were performed.^7^ Continual monitoring of the prostate, with a refresh rate of 25 Hz, is possible with the Calypso tracking system (Varian, Palo Alto, CA). It has been shown that PTV margins as low as 3 mm can be utilized for prostate treatments with the Calypso system.^8^ When used in combination with endo‐rectal balloons (ERBs) that limit the prostate motion, the PTV expansion can be reduced to 2 mm.^9^ Continual monitoring of the prostate position is also possible utilizing MRI Linacs, with a typical image refresh rate of ∼4 Hz. The MIRAGE trial recently utilized 4 mm margins with CT based imaging and no motion tracking compared to MRI based setup and gating, with a 2 mm margin.^10^

Motion measurement is possible utilizing high density fiducials placed within the prostate that can be detected on planar imaging during treatment delivery. The Synchrony system available for use with CyberKnife and Radixact Tomotherapy treatment delivery modalities (Accuray Incorporated, Sunnyvale, CA) can detect the fiducial position during treatment and actively adapts the plan delivery to correct for variation in target position or halt the delivery when large motions are detected.^11^ Fiducial detection can be utilized on C‐arm Linacs to automatically detect the fiducial position at given gantry or time intervals based on user settings and pause the delivery if offsets greater than the user defined tolerance are measured. One example of this is the “Marker Match” feature available with the Edge and Truebeam Linacs (Varian Medical Systems, Palo Alto, CA). Gottschalk and colleagues^12^ determined that for CyberKnife Synchrony treatments of 90–120 min, 2 mm PTV margins would ensure that 90% of patients would receive at least 95% prescribed dose to the CTV. Since the time of this study, CyberKnife treatment times have been greatly reduced and typical times for prostate SBRT are now 30–45 min.^13^ Treatment times with the Radixact Synchrony system and C‐Arm Linacs are shorter still, with typical beam on times of approximately 10 and 5 min, respectively.

To ensure motions measured with the Radixact Synchrony system are representative of prostate motions measured with high frequency techniques, delivery log files of measured target offsets were analyzed. To investigate the dosimetric accuracy of prostate SBRT with both motion compensation and gating techniques five patient plans were delivered using four different motion types utilizing a 3D motion phantom. Due to the relatively slow positional changes seen in prostate SBRT, a longer imaging period should still be sufficient to detect dosimetrically significant prostate motions. We propose that, when intra‐fraction motion is accounted for, adequate CTV coverage can be maintained with PTV margins reduced to 2 mm in all directions.

## 2 METHODS AND MATERIALS

### 2.1 Instrumentation

To allow for dosimetric measurements a Delta4 phantom and Hexamotion motion platform (ScandiDos, Sweden) were utilized. The Delta4 phantom can be manufactured with a removable cubic section that allows for the placement of various tracking objects. In this study a 3D printed cube was utilized containing three 5 mm length, 1 mm diameter, platinum “Visicoil” fiducials (IZI Medical, Owings Mills, MD). In addition to the fiducials, the cube insert was designed to allow for placement of Gafchromic EBT3 film (Ashland, Bridgewater, NJ) both within and adjacent to the cube. This allows for high resolution dosimetric measurements within the PTV, in addition to the measurements acquired with the Delta4's diode arrays, which have a 0.5 cm spacing in the central 6 cm x 6 cm region of the array, and a 1 cm spacing outside of this region.

**FIGURE 1:**
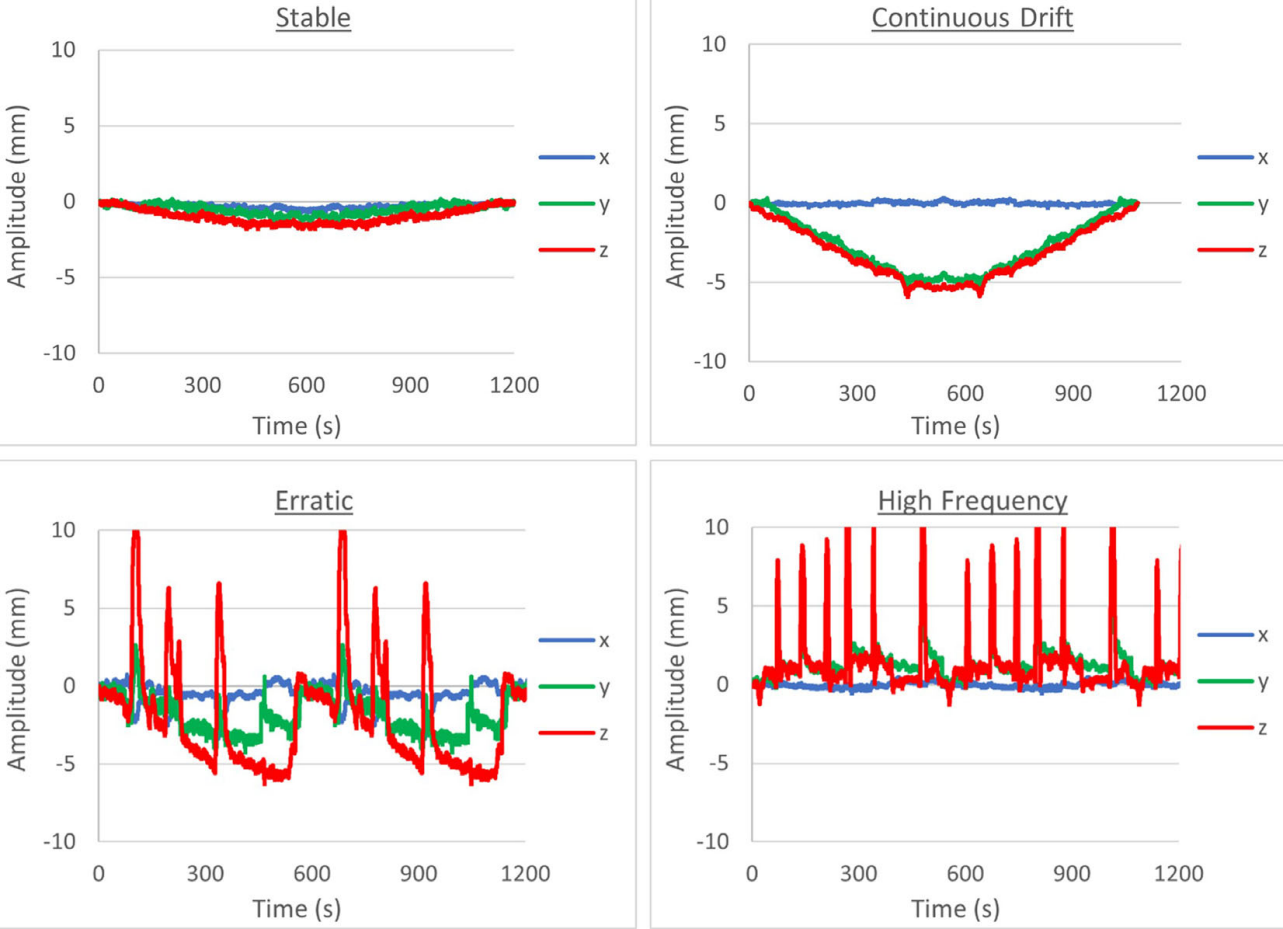
Prostate motion files utilized for phantom motion

When mounted to the Hexamotion platform the Delta4 phantom can be moved with 6 degrees of freedom. In this study only translational shifts were utilized. Four motion files were utilized that represent a range of motions typically seen in prostate treatments^14^ as shown in Figure 1. These motion files are modified from a publicly available dataset^15^ originally measured using the Calypso system (Varian, Palo Alto, CA) acquired at a rate of 25 Hz.

### 2.2 Motion traces

As is typical in intrafraction motion of the prostate, motion is mostly in the anterior/posterior (IEC Z) and inferior/superior direction (IEC Y) with little motion seen in the left/right (IEC X) direction. The “Stable” motion file is a slowly drifting motion with no motion larger than 2 mm. “Continuous Drift” is a slowly drifting motion with a maximum magnitude of 6 mm. “Erratic” is a combination of drifting and large motions. “High Frequency” contains frequent, rapid, large motions. The original motion files^15^ were adjusted to a maximum 10 mm amplitude, from the originals which had a maximum 14.5 mm amplitude. Errors were found with the Hexamotion device and the original files which interrupted the motion delivery. Motion files were also mirrored to increase motion file length.

To ensure the motion files utilized were representative of patient motions measured by the Radixact, log files were extracted for the first 15 patients treated with the system. Seventy‐five deliveries were classified based on the maximum motion offsets detected and the maximum offset gradient. Each delivery was classified as “Stable” with no motion larger than 2 mm, “Drifting” with motions larger than 2 mm and maximum gradient changes less than 0.5 mm per second. Motions with gradient changes larger or equal to 0.5 mm per second were classified as abrupt, these motions are comparable to the “Erratic” and “High Frequency” motion types. Abrupt motions were classified as “Small” where maximum offsets were less than 5 mm and “Large” where maximum offsets were larger or equal to 5 mm.

### 2.3 Plan characteristics

**FIGURE 2:**
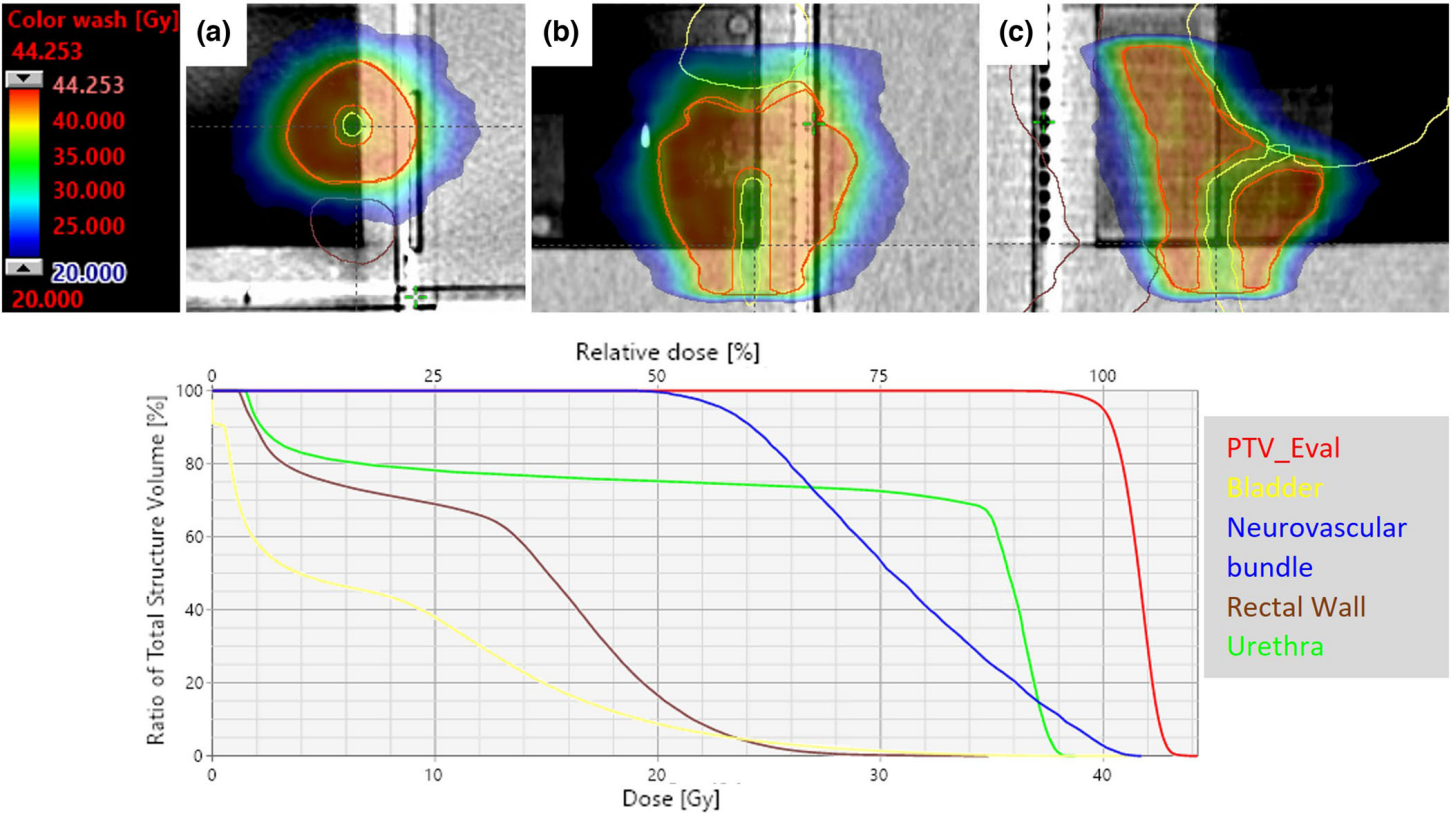
Dose distributions and DVH for one example Truebeam plan showing the axial (a), coronal (b), and sagittal (c) film measurement planes

To allow for examination of the effects of motion on different dynamic MLC patterns/sinograms five patient specific target and OAR volumes were transferred to phantom image sets such that the fiducials were within the PTV and Gafchromic film was positioned within the target volume. Treatment plans were then generated on the phantom CT that met institutional organ at risk (OAR) constraints and conformality requirements with a prescription dose of 40 Gy delivered in five fractions. Figure 2 shows the dose distribution and DVH for one patient plan generated for the Truebeam. Each view represents one of the measurement planes at which Gafchromic film was placed.

OAR overlap of the PTV and bladder, neurovascular bundle, rectum and SpaceOAR hydrogel (Boston Scientific, Natick, MA) was measured for each patient for both the original PTV and the reduced PTV expansion. The original PTV expansion is 5 mm in all directions, except posteriorly where a 3 mm expansion was utilized. For the reduced PTV expansion a 2 mm expansion is used in all directions. SpaceOAR gel is utilized to increase the distance between the PTV and the rectum resulting in small overlap volumes between the rectum and PTV. Overlap with the PTV and SpaceOAR gel was measured to represent potential rectal overlap for patients for whom SpaceOAR is not utilized.

### 2.4 Motion compensation technique

For evaluation of fiducial‐based compensation techniques plans were generated for a Radixact (Accuray Incorporated, Sunnyvale, CA) using a Helical IMRT technique. Each plan utilized a pitch of 0.18, 2.5 cm jaw width and 6 MV flattening filter free (6FFF) energy with a dose rate of 1000 MU/min. The Radixact Synchrony system acquires images of the target at set imaging angles as the gantry rotates around the patient. Measurement of fiducial position versus planned position is calculated with each image and offsets of the jaw and MLC positions are applied until the next image is acquired using the “non‐respiratory” quasi static model.^16^ Four imaging angles were acquired over the course of each gantry rotation at 90‐degree intervals, resulting in an average imaging interval of 8.2 s (7.2– 9.0 s). While the Synchrony system does account for more gradual shifts any large magnitude and rapid position changes will result in treatment delivery being paused depending on the user settings. For this study the “Potential Difference” threshold was set to 4 mm, “Rigid Body” threshold was set to 1.5 mm and “Target Offset” threshold set to 20 mm. After treatment delivery is halted, the user is required to acquire new kV planar imaging to ensure motion has returned to within the set limits before continuing treatment. For real patient deliveries repeat 3D imaging may be required to adjust the patient position, however, in this study as the motions were known this was not necessary. No attempts were made to synchronize the start of motion with the start of treatment as this is most representative of variations in actual patient treatments.

### 2.5 Gating technique

For evaluation of fiducial‐based gating techniques plans were generated for a Truebeam (version 2.7, Varian Medical Systems, Palo Alto, CA) using a VMAT technique. Each plan utilized three arcs and 6FFF energy with a maximum dose rate of 1400 MU/min. The center of each fiducial is denoted as a “marker” in the plan. At the time of delivery, the user is able to acquire kV planar images at fixed gantry, MU, or time intervals. The user sets a given diameter of detection and if any detected fiducial position is outside of this range delivery is paused, the system continues to acquire images at the given time interval and will automatically continue beam delivery once the fiducials are within this range. For this study a 10 s imaging period and 4 mm detection diameter were utilized. In real patient deliveries orthogonal image pairs or 3D imaging may be necessary when patient fiducial positions do not return to within tolerance to adjust the patient position. Since the motions in this study are known, when any motions that would result in a continuous position difference greater than 2 mm were detected, the motion was restarted, resulting in the same effective motion as if the patient had been repositioned.

### 2.6 Data analysis

For both compensation and gating techniques plans were delivered to the phantom in a stationary position as well as for each of the four motion types with and without compensation/gating enabled. Gamma criteria of 3%, 2 mm and 80% threshold dose (relative to prescription dose) were utilized for film analysis. Due to the film placement within the PTV, 80% of prescription dose was chosen as the threshold to focus on achieved gamma pass rates in the high dose region. 3%, 2 mm and 10% threshold dose criteria were utilized for Delta4 diode analysis. Due to the PTV not overlapping the diode arrays a 10% threshold dose was utilized. In each case comparison was made between the measurements when the phantom was stationary and the given motion type.

## 3 RESULTS

Of 75 treatment log motion traces analyzed, 20 were classified as stable, 28 as drifting and 27 as abrupt. Of the abrupt motions 10 were classified as small magnitude and 17 as large magnitude. Table 1 shows the motion classifications for the initial fifteen patients treated with the Radixact Synchrony system. Of the patients analyzed one was found to have large abrupt motions at every fraction. All other patients were found to have daily variability in the type of prostate motions measured. Table 2 shows the measured motion characteristics for the analyzed motion traces.

**TABLE 1:** Motion classification for fifteen patients where “S” = stable, “D” is drifting and “As” and “Al” are abrupt small and large respectively

	Patient														
Fraction	1	2	3	4	5	6	7	8	9	10	11	12	13	14	15
1	D	D	Al	Al	D	S	Al	S	S	S	D	D	D	D	As
2	As	S	Al	Al	D	As	D	As	D	D	As	S	S	S	D
3	As	As	Al	S	As	D	Al	S	D	D	S	D	D	S	D
4	Al	D	Al	S	S	S	Al	Al	D	D	D	D	S	Al	Al
5	D	S	Al	D	D	As	D	Al	S	D	As	S	Al	S	As

**TABLE 2:** Motion characteristics for prostate SBRT measured with the Radixact Synchrony system

	ΔX (mm)		ΔY (mm)		ΔZ (mm)	
Motion type	Mean (St. Dev)	Maximum	Mean (St. Dev)	Maximum	Mean (St. Dev)	Maximum
Stable	0.9 (0.2)	1.4	1.5 (0.3)	1.9	1.4 (0.3)	1.9
Drifting	1.2 (0.6)	3.5	2.6 (1.4)	6.5	3.0 (1.5)	7.1
Abrupt small	1.4 (0.4)	2.1	3.3 (0.7)	4.2	2.6 (1.2)	4.3
Abrupt large	4.8 (4.2)	15.1	6.7 (3.5)	15.3	9.4 (4.5)	18.1

Overlap of the PTV with nearby OAR's was measured for the bladder, neurovascular bundle, rectum, and SpaceOAR gel as shown in Table 3. For the patient targets used in this study a median prostate volume of 38.7 cm^3^ (30.4–99.6 cm^3^) was measured. Final CTV volume, with seminal vesicle inclusion based on individual patient disease, had a median value of 49.7 cm^3^ (35.9–107.0 cm^3^). Original PTV volumes utilizing a 5 mm expansion in all directions, except posteriorly where a 3 mm margin is utilized, resulting in a median PTV volume of 103.6 cm^3^ (73.6–179.4 cm^3^). With the margin reduced to 2 mm in all directions this was reduced to 75.3 cm^3^ (53.3–138.1 cm^3^)

**TABLE 3:** Organ at risk and PTV overlap volume

Organ at risk	Original PTV Overlap Volume (cm^3^)	Reduced PTV Overlap Volume (cm^3^)	Volume reduction
Bladder	5.5	1.6	74%
Neurovascular bundle	1.8	0.7	63%
Rectum	0.3	0.0	96%
SpaceOAR	3.1	1.3	61%

Table 4 shows the average film gamma pass rate for the five delivered patient plans using a 3%, 2 mm and 80% threshold dose (relative to the prescription dose). In each case comparison was made between the measured films when the phantom was stationary and the given motion type. Film measurements for the Radixact system had an average pass rate for uncorrected motions of 80.5%. Corrected motion results in a pass rate of 96.0%. For the Truebeam system an average pass rate of 89.9% was found for uncorrected motions measurements. Gated motion results in a pass rate of 94.8%.

**TABLE 4:** Average gamma pass rates for Gafchromic film measurements using a gamma criteria of 3%, 2 mm, 80% threshold dose for uncorrected and corrected/gated measurements with the Radixact and Truebeam systems respectively

Motion type	Radixact synchrony		Truebeam marker match	
	Uncorrected (St. Dev)	Corrected (St. Dev)	Uncorrected (St. Dev)	Gated (St. Dev)
Stable	98.6% (1.2%)	98.9% (0.9%)	93.7% (12.2%)	99.7% (0.2%)
Drifting	70.2% (9.2%)	95.4% (2.7%)	85.2% (11.2%)	87.7% (14.8%)
Erratic	73.2% (6.4%)	95.4% (2.1%)	92.1% (5.1%)	94.2% (11.0%)
High frequency	80.1% (4.8%)	94.3% (2.9%)	88.4% (9.6%)	97.6% (2.4%)

Table 5 shows the average Delta4 gamma pass rates for five delivered patient plans using a 3%, 2 mm and 10% threshold dose (relative to the prescription dose). Delta4 diode arrays measurements for the Radixact system had an average pass rate for uncorrected motions of 83.8%. Corrected motion results in a pass rate of 94.8%. For the Truebeam system an average pass rate of 87.2% was found for uncorrected motions measurements. Gated motion results in a pass rate of 96.9%.

**TABLE 5:** Average gamma pass rates for Delta4 diode measurements using a gamma criteria of 3%, 2 mm, 10% threshold dose for uncorrected and corrected/gated measurements with the Radixact and Truebeam systems, respectively

Motion type	Radixact Synchrony		Truebeam Marker Match	
	Uncorrected (St. Dev)	Corrected (St. Dev)	Uncorrected (St. Dev)	Gated (St. Dev)
Stable	99.7% (0.2%)	100.0% (0.1%)	99.9% (0.2%)	99.8% (0.5%)
Drifting	73.5% (6.1%)	90.0% (4.7%)	81.7% (5.5%)	99.7% (0.4%)
Erratic	77.9% (2.5%)	95.3% (1.7%)	87.1% (4.2%)	98.0% (1.9%)
High frequency	84.1% (3.2%)	93.7% (3.8%)	80.1% (4.8%)	90.2% (5.8%)

## 4 DISCUSSION

Analysis of prostate motions calculated by the Radixact synchrony system shows similar patterns as those previously reported and utilized in this study. The source of the motion, be it either internal motion or patient motion is not possible to determine. Of the 15 initial patients treated with the system, all patients demonstrated motions larger than 2 mm in at least one fraction. Large motions resulted in both the Synchrony and Marker Match systems pausing the treatment delivery. For patients who demonstrate frequent shifts leading to interruptions the total treatment time can be significantly increased. For such patients a larger PTV margin and less stringent tracking parameters might be required, however, measurements of overlap volumes with OARS adjacent to the PTV clearly indicate the potential reduction of high dose to these OARs when a smaller PTV margin is utilized.

For Gafchromic film measurements similar pass rates were seen for both Truebeam and Radixact uncorrected deliveries, with Radixact having a lower average pass rate, possibly due to the increased treatment time. Truebeam deliveries were found to have a blurring of the delivered isodose distribution, whereas more distinct shifts were seen in Radixact deliveries. Drifting and erratic motions were found to show shifts in the Radixact sagittal and coronal films, which, due to the helical nature of the Radixact delivery, could be seen to increase over time and matched the offset of the motion file.

Radixact deliveries had a lower standard deviation for the majority of both uncompensated and compensated deliveries. It has been demonstrated in previous work for lung treatments^17^ that the binary MLCs and helical delivery are less sensitive to MLC interplay effects, for sinusoidal motions, than VMAT deliveries. Although the motion files utilized in this study are not sinusoidal, interplay effects can still exist, and have been demonstrated in prostate treatments using VMAT.^18^ The higher standard deviations shown in the VMAT deliveries in this study indicate that a similar trend may exist for helical tomotherapy and VMAT deliveries for prostate motions.

For corrected deliveries the Radixact Synchrony system showed average gamma pass rates greater than 90% with standard deviations less than 3% for all motion types. Gated deliveries with marker match on the Truebeam also showed high pass rates, with only the gradual drift motion showing an average pass rate below 90%. Again, higher standard deviations were seen in the gated Truebeam deliveries than in the corrected Radixact deliveries. Delta4 planar diode measurements support these film measurements with average pass rates for all corrected and gated deliveries greater than, or equal to 90%.

Levin‐Epstein et al.^19^ estimated a minimum PTV margin of 4 mm was required in the absence of intra‐fraction motion management, noting a 1–2 mm margin was required for systematic errors in target contouring and machine performance, when fiducials are utilized. A recent study of prostate SBRT with the CyberKnife Synchrony system by Gupta et al.^20^ analyzed prostate motion during treatment delivery and calculated the PTV margins required to account for this intrafraction motion. The study also included a review of multiple contemporary studies and the various prostate motions and PTV margins utilized. While Yu et al.^21^ determined that margins of 2.7 mm were required to ensure 95% CTV coverage, again for CyberKnife treatments. Aubrey et al.,^22^ van de Water et al.^23^ and Wu et al.^24^ all determined that 3 mm PTV margins ensured 98% CTV coverage in the majority of treatment sessions. Uninterrupted prostate SBRT treatments with C‐arm Linacs and the Radixact system typically have beam times of 5–10 and 10–15 min, respectively. These treatments times are much shorter than CyberKnife treatments and multiple studies have shown that the longer the treatment time, the larger magnitude motion shifts,^25^, ^26^ although studies have noted a plateau in motion magnitude after approximately 20 min.^27^

In this study imaging intervals were relatively short when compared to the imaging intervals of previous CyberKnife studies. It has been shown that rapid prostate motions can occur, so the authors believe these shorter imaging intervals are warranted to allow for treatment to be halted after large shifts have occurred. While it is still possible for large transient shifts to occur between successive kV verification images even with short imaging intervals, the excursion caused by these shifts would be so rapid that there will likely be little dosimetric consequence. The “Continual Drift” and “Erratic” motion types were found to cause the lowest pass rates when no compensation or gating was applied. The mean target offset in the motion file was found to have a larger impact than the maximum offset, as noted previously, large magnitude shifts were typically short in duration.

Although real time tracking is possible with devices such as MRI Linacs or the Calypso system, MRI Linacs remain a relatively rare treatment modality and the transponders utilized in the Calypso system limit the possibility of MRI imaging for target delineation. Calypso transponders cause a “dead zone” in MRI imaging where no signal is measured. This artifact can be up to 2 cm in radius and 3 cm in length^28^ per transponder and can be larger than the prostate itself. Studies have shown that the prostate gland is typically overestimated by as much as 35% when contoured on CT images as compared to MRI imaging,^29^ therefore, MRI imaging is essential for target delineation in SBRT. While delivery accuracy could be increased using Calypso for continual target tracking, OAR overlap volume may be increased, due to this additional uncertainty in CTV delineation. The authors had originally intended to utilize the Calypso system for comparison, however, the Delta4 phantom was found to interfere with the Calypso detector array, preventing its use.

Inter‐fraction variations in prostate and proximal (1 cm) seminal vesicle rotations have been demonstrated with MRI Linacs^30^ and whilst prostate rotations were found to be minimal, seminal vesicle rotations were found to be significant. Pre‐treatment 3D volumetric imaging is essential to SBRT, gross target volume should be examined, in addition to fiducial based localization, and where large changes are seen the patient should not be treated at that time. The same study also showed prostatic volume increased over the course of treatment for all patients, highlighting the potential need for replanning CTs, or perhaps adaptive planning, when such tight PTV margins are utilized.

This study considers translation shifts only and does not consider rotational changes in the target position, since the Radixact system is only capable of applying translational corrections. Intra‐fraction rotational changes of the prostate have been demonstrated by Wolf et al.,^31^ with pitch rotations being demonstrated as occurring in the majority of patients and having the largest dosimetric impact. Their work noted that translational shifts were sufficient to account for rotational uncertainties, however, larger margins were utilized. Their work does specify that a 3 mm margin was sufficient to ensure dosimetric coverage of the CTV and that a 3 mm uniform margin would be utilized by the group in future studies.

Whilst this study does not investigate the effects of motion changes on DVH metrics, the potential sparring of high dose to nearby OARs is demonstrated in the reduced overlap volumes. Larger margins, which ensure CTV coverage when motion is not considered, can be significantly reduced when motion is taken into account.

## 5 CONCLUSION

These results show that very high gamma pass rates are achievable when motion compensation and gating techniques are applied for prostate SBRT. With these techniques a 2 mm PTV margin, that takes imaging and delivery uncertainties into account, seems to be sufficient to ensure adequate CTV coverage. When using such small margins, it is critical that pre‐treatment volumetric imaging is utilized to ensure there are no anatomical changes, such as seminal vesicle rotations and prostatic volume increases. The reduction of PTV margins, and the potential decreases in patient toxicities must be offset against the possibility of patients not undergoing treatment due to these anatomical changes, and increased numbers of replans.

## AUTHOR CONTRIBUTIONS

All authors had significant input in the design of the work, revision, and final approval of the work. Lee Goddard was responsible for collection and analysis of data. All authors agree to be accountable for all aspects of the work in ensuring that questions related to the accuracy or integrity of any part of the work are appropriately investigated and resolved.

## CONFLICTS OF INTEREST

Montefiore Medical Center has ongoing collaboration agreements with Accuray Inc. Dr's Garg and Tomé have previously received research funding from Accuray Inc. and Varian Medical Systems not related to this work.
